# Developing and applying a gene functional association network for anti-angiogenic kinase inhibitor activity assessment in an angiogenesis co-culture model

**DOI:** 10.1186/1471-2164-9-264

**Published:** 2008-06-02

**Authors:** Yuefeng Chen, Tao Wei, Lei Yan, Frank Lawrence, Hui-Rong Qian, Timothy P Burkholder, James J Starling, Jonathan M Yingling, Jianyong Shou

**Affiliations:** 1 Angiogenesis and Tumor Microenvironment Biology, Lilly Research Laboratories, Indianapolis, IN 46285, USA; 2 Integrative Biology, Lilly Research Laboratories, Indianapolis, IN 46285, USA; 3 Statistics, Lilly Research Laboratories, Indianapolis, IN 46285, USA; 4 Discovery Chemistry, Lilly Research Laboratories, Indianapolis, IN 46285, USA; 5 Current Address: China Novartis Institutes for BioMedical Research, Shanghai 201203, PR China

## Abstract

**Background:**

Tumor angiogenesis is a highly regulated process involving intercellular communication as well as the interactions of multiple downstream signal transduction pathways. Disrupting one or even a few angiogenesis pathways is often insufficient to achieve sustained therapeutic benefits due to the complexity of angiogenesis. Targeting multiple angiogenic pathways has been increasingly recognized as a viable strategy. However, translation of the polypharmacology of a given compound to its antiangiogenic efficacy remains a major technical challenge. Developing a global functional association network among angiogenesis-related genes is much needed to facilitate holistic understanding of angiogenesis and to aid the development of more effective anti-angiogenesis therapeutics.

**Results:**

We constructed a comprehensive gene functional association network or interactome by transcript profiling an in vitro angiogenesis model, in which human umbilical vein endothelial cells (HUVECs) formed capillary structures when co-cultured with normal human dermal fibroblasts (NHDFs). HUVEC competence and NHDF supportiveness of cord formation were found to be highly cell-passage dependent. An enrichment test of Biological Processes (BP) of differentially expressed genes (DEG) revealed that angiogenesis related BP categories significantly changed with cell passages. Built upon 2012 DEGs identified from two microarray studies, the resulting interactome captured 17226 functional gene associations and displayed characteristics of a scale-free network. The interactome includes the involvement of oncogenes and tumor suppressor genes in angiogenesis. We developed a network walking algorithm to extract connectivity information from the interactome and applied it to simulate the level of network perturbation by three multi-targeted anti-angiogenic kinase inhibitors. Simulated network perturbation correlated with observed anti-angiogenesis activity in a cord formation bioassay.

**Conclusion:**

We established a comprehensive gene functional association network to model in vitro angiogenesis regulation. The present study provided a proof-of-concept pilot of applying network perturbation analysis to drug phenotypic activity assessment.

## Background

Angiogenesis, the generation of new blood vessels, plays an essential role under normal physiological conditions as well as during the pathogenesis of many diseases including atherosclerosis, macular degeneration, wound healing, diabetic retinopathy, and human malignancy [1-3]. Remarkably, tumor dormancy is believed to be attributed, at least in part, to the lack of angiogenesis support. The transition from an avascular, dormant tumor to an aggressively growing angiogenic cancer is referred to as the "angiogenic switch" [4,5]. More than 30 years ago, it was hypothesized that inhibition of tumor angiogenesis would inhibit solid tumor growth [6]. Since then, the expansion of angiogenesis research has resulted in the identification of various pro- and anti-angiogenic factors, such as FGF, VEGF, angiopoietin, endostatin, vasostatin, and neuronal cell axon guidance molecules [2,7-9] and the development of several anti-tumor angiogenesis medicines that have recently proven efficacious in the clinic [1,2,7,10,11].

Malignant tumors often express an array of angiogenic factors to potentiate angiogenesis and tumor growth [8]. Ultimate angiogenic outcomes depend on the dynamic equilibrium between positive and negative regulators and the interplay among their signal transduction pathways, not on a single discrete pathway. Avastin, a monoclonal antibody that specifically blocks VEGF, is the first approved anti-angiogenic therapy. Notably, combination with chemotherapies has been necessary for its clinical efficacy. Moreover, tumors often develop drug resistance in response to anti-VEGF therapy [12]. Hanahan et al demonstrated one possible mechanism by which such drug resistance might develop[13]. Inhibition of VEGF signalling by neutralizing antibody to VEGFR2 (KDR) induces elevated expression of hypoxia associated proangiogenic factors such as FGF and EphrinA1, which subsequently reactivates VEGF independent angiogenesis and tumor growth. Thus, disrupting a single proangiogenic pathway by itself is often insufficient to achieve sustained therapeutic benefits. In this light, it is necessary to explore global functional association among angiogenesis-related genes rather than focusing on an individual or a few angiogenesis factors discretely.

Importantly, angiogenesis involves intercellular interaction among vessel-forming endothelial cells, nonmalignant cells such as the supporting pericytes, immune and stromal cells, as well as the malignant tumor cells [1,14]. Therefore, global gene-gene interactions during angiogenesis need to be explored in a multi-cell type context. Human umbilical vein endothelial cells (HUVECs) are widely used to study vascular biology [15]. They form a lumen bearing capillary structure when co-cultured with normal human dermal fibroblast (NHDF) cells [16]. It has also been noticed that HUVECs change their cellular and molecular properties upon passage *in vitro*, a phenomenon thought to be due to *in vitro *cellular senescence [17-20]. In the present study, we determined that HUVEC competence and NHDF supportiveness for angiogenesis in this co-culture system are both cell passage dependent. Gene Ontology (GO) analysis of differentially expressed genes showed that the cell passage dependent global transcriptional changes are highly related to angiogenesis. This enabled us to construct a comprehensive functional association network of differentially expressed genes using a natural language processing algorithm. We further developed a "network walking" algorithm to estimate network perturbation by small molecule kinase inhibitors. The simulated compound activity via network perturbation analysis was in good agreement with actual phenotypic activity in the cord formation bioassay.

## Results

### Characterization of an *in vitro *angiogenesis co-culture model by multi parameter high content image analysis

**Figure 1:**
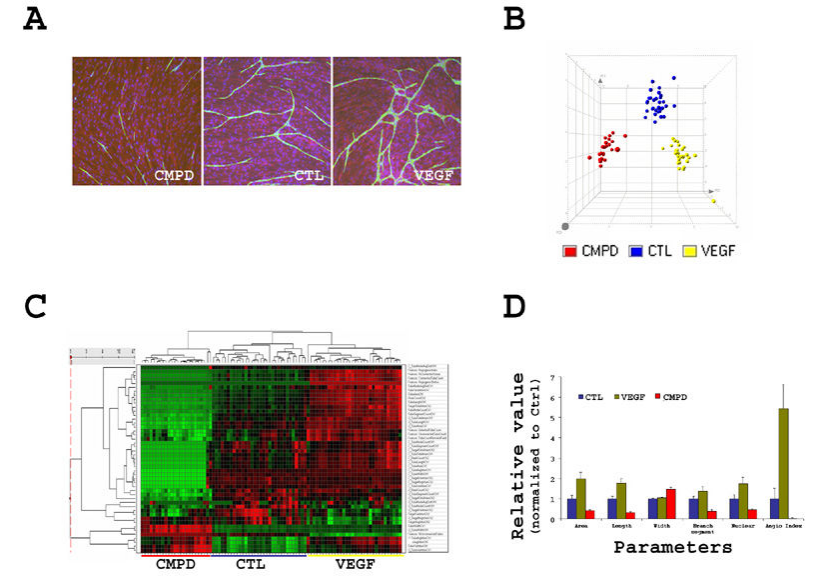
**Multi parameter characterization of *in vitro *angiogenesis in a co-culture model**. **(A) **HUVECs formed capillary structure (cord) when plated on dermal fibroblast cells over the period of 12–14 days. The angiogenic cord structure was visualized by human CD31 staining (green), fibroblast cells were stained with an intermediate filament marker vimentin (red), while the nucleus of both cell types were visualized with Hoechst staining (blue). Cord formation was greatly enhanced by addition of 20 ng/ml VEGF (VEGF) to the culture, while 30 nM Sunitinib (CMPD) drastically inhibited cord formation as compared to the medium control (CTL). **(B)**. Morphological features of the cord formed in the co-culture were captured and quantitated by high content image analysis using the ArrayScan platform adopting the tube formation bioapplication. 47 measurements were assessed, normalized using the Z-score across different treatment groups and analyzed by Principal Component Analysis (PCA). The first three principal components accounting for more than 80% of the variance were selected for sample scatter plotting. Blue are the control cultures (CTL); yellow, cultures treated with 20 ng/ml VEGF; and red, cultures treated with 30 nM Sunitinib (CMPD) treated samples. **(C)**. The Z-scores of 47 morphological measurements were clustered in Spotfire Decision Site with the correlation coefficient being used as the distance metric. Complete linkage was used as the clustering algorithm. Red stands for higher and green for lower than mean (black) expression. **(D)**. Six representative parameters were selected and plotted. Except for the cord width, the cord length, area, branching point, the number of nucleus within the cord, and a composite angiogenesis index (Angio Index) all manifested the anti and pro angiogenic effects from Sunitinib and VEGF, respectively. Data are expressed as Mean ± S.D.

HUVECs form a capillary structure when co-cultured with NHDF *in vitro *[16]. Unlike the HUVEC Matrigel™ assay, the HUVEC/NHDF cord formation co-culture allows us to study cell-cell interactions during long term (up to 14 days) angiogenesis. The formation of the cord structure is a highly organized morphogenic process. We developed a high content imaging based assay to document and quantitate cord formation using the Cellomics ArrayScan platform. We were able to simultaneously assess dozens of morphological measurements, such as tube length, area, connectivity of the cord, branching, and number of nuclei within the cord. Using this assay system, we tested a clinically approved antiangiogenic compound Sunitinib (SUTENT, SU11248). While 20 ng/ml VEGF significantly increased angiogenesis, 30 nM Sunitinib significantly inhibited angiogenesis (Fig. 1A).

To explore the phenotypic signatures induced by VEGF or Sunitinib, we performed principal component analysis (PCA) and hierarchical clustering (Fig. 1B &1C). Multivariate analyses using 47 morphological measurements clearly distinguished the three treatment groups, i.e. control, VEGF, and Sunitinib. In the PCA analysis, the first three principal components captured more than 80% of the total variance. Examining the PC loadings, we found that among the most discriminative features are the cord length (either connected or unconnected or in general), cord area, the branching segment count. The cord CD31 immunofluorescence intensity is also one of the features that significantly contributed to PC1, likely due to the overall correlation of fluorescence staining to cord mass. Interestingly, the cord width is not a discriminative feature for either VEGF or Sunitinib action. The current multi-parameter, high content analysis allowed for a detailed dissection of the phenotypic characteristics associated with a given factor or compound. Shown in Fig. 1D is the mean value plot of a few representative parameters. Sunitinib significantly inhibited the mass of the cord in length and area, but not width. Moreover, the number of nuclei per tube is significantly reduced, indicating that Sunitinib could have anti-proliferation activity on HUVECs in the co-culture. Sunitinib treated cultures are less morphologically complex, as the number of connected cords, the branching nodes, and segment measurements are all significantly reduced.

### HUVEC cord formation competence is cell passage dependent

**Figure 2:**
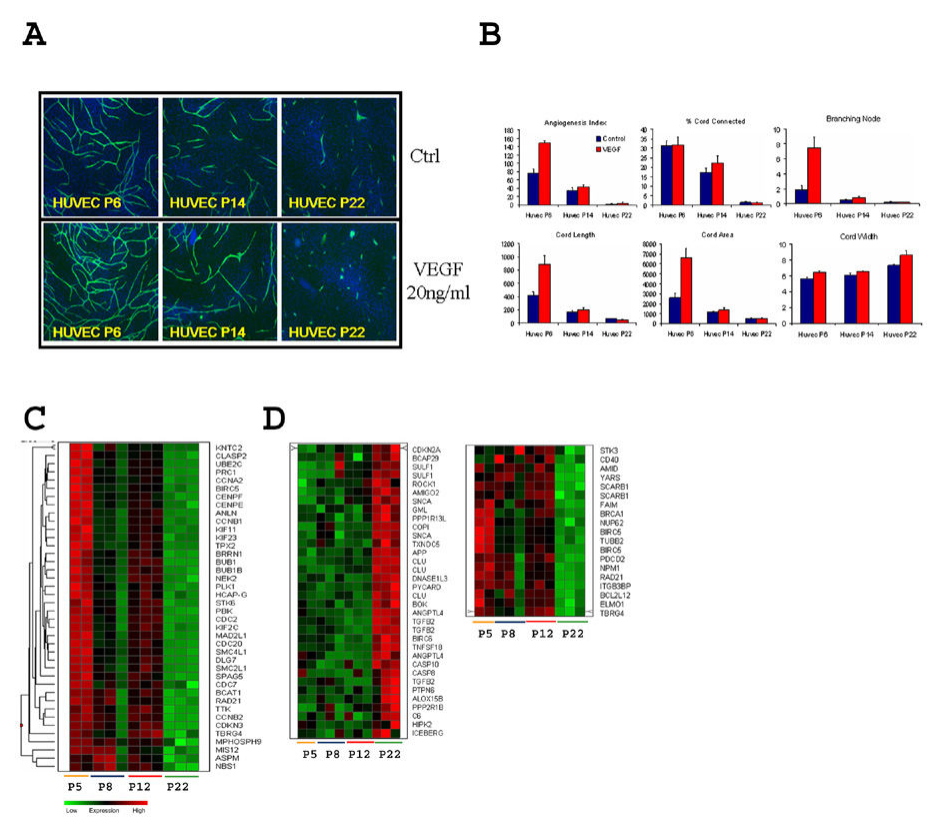
**HUVEC passage dependent angiogenic competence in cord formation assay**. HUVECs cultured to various passage numbers (P6, early; P14, intermediate; and P22, late) were co-cultured with NHDFs in the cord formation assay. The cord was visualized by human CD31 staining (green) and quantitated. The cultures were treated with or without 20 ng/ml exogenously added VEGF. Representative images are shown in (**A**). 6 well-level parameters were quantitated and plotted in (**B**). Please note that HUVECs gradually lost their cord formation competence when passaged *in vitro*. Data are expressed as Mean ± S.D. (**C-D**). HUVECs with various passage numbers (P5, P8, P12, and P22) were assayed for basal gene expression using the Affymetrix GeneChip platform. A linear regression method was used to identify differentially expressed genes over cell passage. An enrichment testing was used to identify significantly changed Biological Process (BP) categories associated with HUVEC passage-dependent gene expression. Representative BP categories were shown using heatmap visualization, for genes associated with mitotic cell cycle (**C**), and cell death (**D**).

It has been well known that HUVECs change their biochemical and cellular behaviors when passaged *in vitro *[17-20]. We cultured HUVEC cells from a single batch to various passages (P6, P14, P22) and tested them for cord formation competence. We found that they gradually lost cord formation capabilities upon passage (Fig. 2A). While early passage (P6) HUVECs formed very nice capillary structures and responded robustly to VEGF when co-cultured with NHDF, late passage (P22) HUVECs lost cord formation capability and had diminished response to VEGF (Fig. 2A &2B).

We next profiled gene expression changes associated with HUVEC passage. Since the loss of angiogenesis capability was proportional to the round of cell passage, we applied a statistical approach to identify genes with regressive expression patterns, i.e. their expression either increased or decreased over passage numbers. We identified 1103 differentially expressed (DE) genes which are listed in Supplemental Table 1 (see Additional file 1). In order to understand the biological relevance of the DE genes, we analyzed the DE genes by using the DAVID functional annotation system [21]. Table 1 lists Gene Ontology (GO) terms significantly enriched with the DE genes. The most significant Biological Processes (BP) categories are cell cycle, proliferation and programmed cell death. Expression profiles of the DE genes involved in representative biological processes were analyzed by hierarchical clustering analysis. The majority of the DE genes involved in mitotic cell cycle and proliferation were down-regulated, whereas the majority of genes related to cell death were up-regulated (Fig. 2C–D), which supports the replicative cell senescence hypothesis.

**Table 1:** Gene Ontology (GO) terms enriched with differentially expressed genes in HUVEC of different passages

**GO^1^**	**GO Term**	**DEG^2^**	**FDR^3^**
Biological Process	cell cycle	93	0.00075
	mitotic cell cycle	44	0.00042
	M phase of mitotic cell cycle	36	0.00124
	programmed cell death	44	0.00717
	mitosis	35	0.00511
	cell division	37	0.00332
	regulation of cell cycle	60	0.01370
	cell cycle checkpoint	13	0.01512
	cell organization and biogenesis	113	0.05000
	cell proliferation	51	0.01164
Molecular Function	protein binding	270	0.00051
	ATP binding	106	0.00048
	nucleotide binding	137	0.00473
	catalytic activity	309	0.00144
	purine nucleotide binding	119	0.00210
	cytoskeletal protein binding	33	0.00545
	kinase activity	70	0.03651
	binding	545	0.03367
	transferase activity	116	0.01551
Cellular Component	spindle	14	0.00058
	microtubule cytoskeleton	36	0.00182
	intracellular	439	0.00138
	cytoskeleton	73	0.00558
	nucleus	237	0.00615
	intracellular membrane-bound organelle	317	0.04183
	intracellular organelle	364	0.01793

1. Gene Ontology; 2. Differentially expressed genes; 3. False discovery rate

Listed are GO categories identified through enrichment testing of the differentially expressed genes from HUVEC using the DAVID functional annotation system as described in the methods section.

### Profiling passage dependent angiogenesis support by dermal fibroblast cells

**Figure 3:**
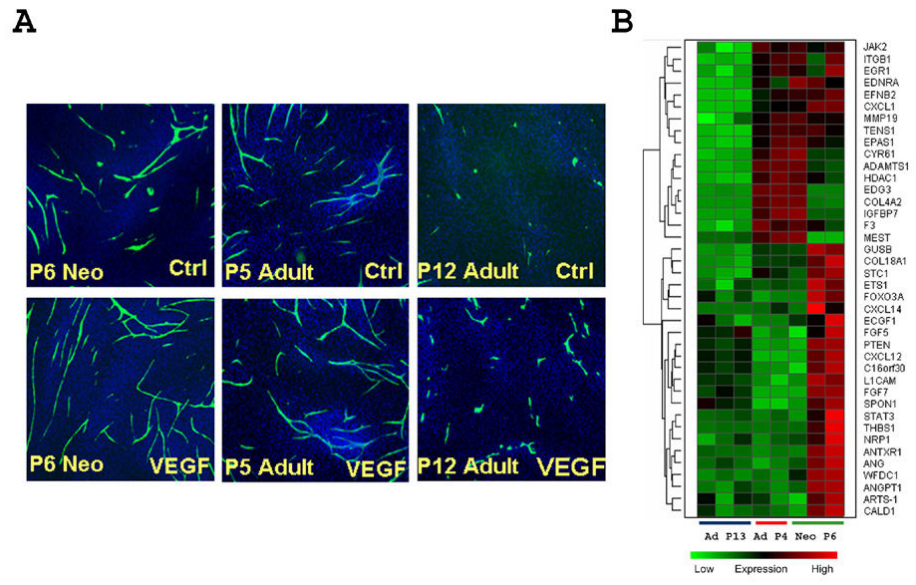
**NHDF passage dependent angiogenic supportiveness in cord formation assay**. P6 NHDFs from a neonatal donor, P5 and P12 NHDFs from an adult donor were tested for their angiogenesis supportiveness in the cord formation assay (**A**). Cultures were performed either with or without 20 ng/ml exogenously added VEGF. Cords were visualized by human CD31 staining (green). Both early passage neonatal and adult NHDF cells are more supportive for cord formation than the late passage adult NHDFs, with the late passage NHDF cells being non-permissive. (**B**). Clustering analysis of genes involved in angiogenesis. P6 NHDFs from a neonatal donor (Neo P6), P4 NHDFs from an adult donor (Ad P4), and P13 NHDFs (Ad P13) from the same adult donor were assayed for basal gene expression using the Affymetrix GeneChip platform. 787 nonredundant genes were identified as common differentially expressed genes between the two early passage NHDF and the late passage NHDF cells. Enrichment testing was used to identify significant Biological Process (BP) categories. Representative angiogenesis BP categories are shown in a heatmap visualization.

Long term sustained angiogenesis in co-culture is supported by dermal fibroblast feeder layer cells, which are also affected by cell passage. While late passage (P12) adult NHDFs demonstrated poor support for HUVECs to form capillary structures, early passage (P5) adult cells did well (Fig. 3A), suggesting that supportiveness of NHDFs in the angiogenesis assay is indeed cell-passage dependent. An early passage NHDF from a neonatal donor also supported angiogenesis in this assay (Fig. 3A).

Differential angiogenesis supportiveness by NHDFs in the co-culture system represents a unique opportunity for us to dissect factors that may contribute to the complex intercellular communications during angiogenesis. To assess this, we profiled gene expression in the three listed preparations of NHDF cells by DNA microarray analysis. Between the supportive NHDF cells versus the non-supportive ones, we identified 787 non-redundant common DE genes which are listed in Supplemental Table 2 (see Additional file 2). Similarly, we used the DAVID functional annotation system to examine the functional relevance to angiogenesis of these DEGs. Table 2 lists the significantly enriched GO terms. Interestingly, blood vessel development and morphogenesis, angiogenesis and vasculature development were among the most significantly enriched biological processes, suggesting that these molecular changes were related to cellular potential to support angiogenesis. Representative expression profiles of genes involved in blood vessel development and angiogenesis are shown in Fig. 3B. This result is consistent with the supporting roles of NHDFs in this co-culture system. Taken together, passaging NHDF cells *in vitro *adversely affects cellular capacity to support angiogenesis due to significantly orchestrated changes of many genes involved in a few related biological processes.

**Table 2:** Gene Ontology terms enriched with differentially expressed genes in NHDF cells of different passages

**GO^1^**	**GO Term**	**DEG^2^**	**FDR^3^**
Biological Process	blood vessel development	64	0.00700
	vasculature development	64	0.00700
	actin filament organization	40	0.00850
	actin cytoskeleton organization and biogenesis	81	0.00850
	actin filament-based process	84	0.00850
	regulation of signal transduction	98	0.01020
	angiogenesis	56	0.01140
	blood vessel morphogenesis	60	0.01140
	cell morphogenesis	161	0.02020
Cellular Component	extracellular matrix	75	0.00200
	proteinaceous extracellular matrix	74	0.00200
	actin cytoskeleton	60	0.04800
	adherens junction	30	0.06140
	cell-matrix junction	25	0.06140
	cytoskeleton	132	0.06140
Molecular Function	protein binding	864	0.00820
	binding	1195	0.01740
	cytoskeletal protein binding	95	0.02380

1. Gene Ontology; 2. Differentially expressed genes; 3. False discovery rate.

Listed are GO categories identified through enrichment testing of the differentially expressed genes from NHDF cells using the DAVID functional annotation system as described in the methods section.

**Figure 4:**
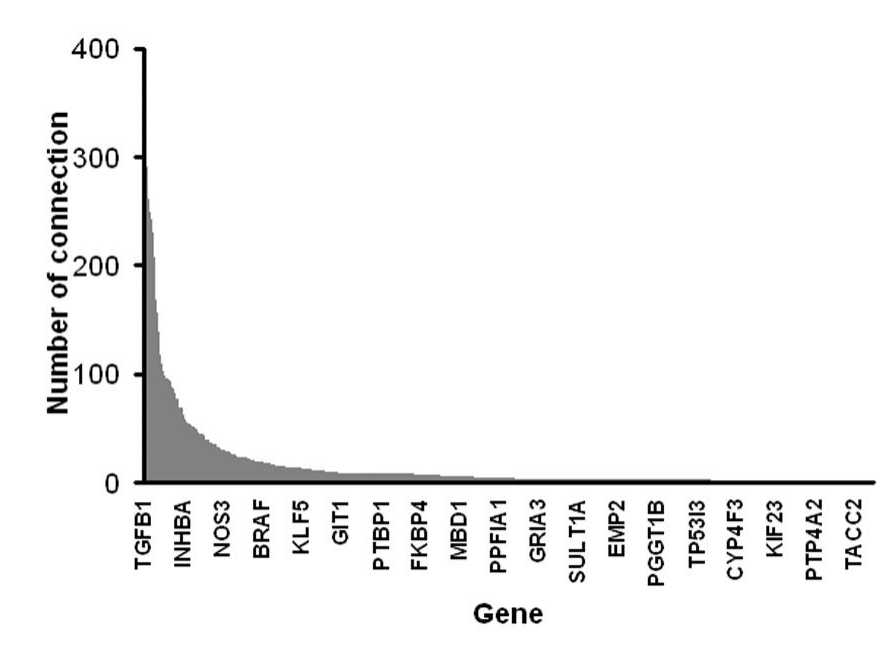
**Interactome analysis of the gene functional association in the HUVEC/NHDF co-culture**. The interactome that was built with PathwayAssist from a union of differentially expressed genes identified in the microarray studies consists of 17226 connections among 1201 genes. The distribution of the number of connections for 1201 genes is sorted by its connectivity, which acts as a typical scale-free network.

Establishment of a gene functional association networkThe fact that cell passage dependent gene expression in both HUVEC and NHDF cells influences angiogenesis supports modeling the angiogenesis network using the DEGs identified by these DNA microarray experiments. To understand the functional association of these genes, we constructed a molecular interactome among the total 2012 differentially expressed genes identified from either NHDFs or HUVECs. The interactome consisted of over 23,000 gene-to-gene connections retrieved from PubMed abstracts using the natural language processing algorithm in PathwayAssist. We then refined the interactome by removing genes whose expression was not detectable by microarray analysis in the co-culture system. The resulted molecular interactome consists of 17226 molecular relationships described by the following terms: binding, expression, molecular transport, protein modification and regulation (see Additional file 3). There were 1201 unique genes in the interactome, among which 887 genes were differentially expressed in the co-culture system. Thus, the interactome covers roughly 74% of the identified DEGs. The interactome displays the typical characteristics of a scale-free network [22] (Fig. 4). Thirty genes with the highest connectivity are listed in Table 3. We further identified 823 angiogenesis-related genes by conducting automated querying of the PubMed abstract database using an in-house developed text mining tool, TargetMiner. The thirty most connected genes in the interactome were all angiogenesis related, accounting for over 33% of the total 17226 connections in the interactome. This data suggests a high degree of relevancy of the interactome to angiogenesis.

**Table 3:** The most connected thirty molecules in the interactome

**Rank**	**Gene**	**Connectivity**	**Percentage**
1	TGFB1	397	2
2	TP53	301	4
3	IGF1	290	6
4	MAPK1	270	7
5	FGF2	261	9
6	PDGF	251	10
7	AKT1	248	12
8	SRC	243	13
9	JUN	242	15
10	AGT	231	16
11	SP1	230	17
12	IL6	220	18
13	IL1B	216	20
14	NGFB	207	21
15	VEGF	183	22
16	MYC	173	23
17	MAPK8	169	24
18	HGF	168	25
19	RAF1	162	26
20	MAPK3	156	27
21	CREB1	146	28
22	EP300	141	28
23	EGFR	138	29
24	CDKN1A	122	30
25	RAC1	117	31
26	STAT3	115	31
27	BCL2	109	32
28	PRL	108	33
29	PPARG	103	33
30	NR3C1	101	34

Genes within the regulation network or interactome were ranked by their connectivity in the network, i.e., the number of genes that they directly engaged with. Listed above are the top 30 genes with the highest connectivity. Gene symbols are those assigned by Human Gene Nomenclature Committee (HGNC).

### Subnetwork analysis of oncogene involvement in angiogenesis

**Figure 5:**
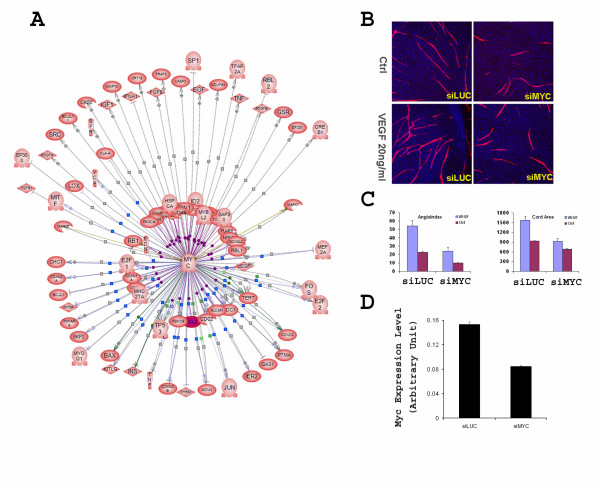
**Analysis of the involvement of MYC oncogene in angiogenesis**. **(A)**. Subnetwork analysis of MYC in the interactome. Differentially expressed genes identified from the HUVEC and NHDF microarray studies were used to build a gene regulation interactome as described in the method section. Genes connected to MYC were extracted from the interactome and visualized by building a MYC centric subnetwork. **(B)**. Validation of the involvement of MYC in cord formation by siRNA mediated gene silencing. Expression of MYC in the HUVECs was attenuated by transfecting the cells with siRNA against MYC (siMYC). The siRNA treated HUVECs were co-cultured with regular NHDF cells. siRNA against luciferase (siLUC) was used as the negative control. Knocking down the MYC expression in HUVECs impaired cord formation either in the presence or absence of 20 ng/ml VEGF. **(C)**. Quantitation of cord area and angiogenesis index from the siRNA experiment. **(D)**. Validation of reduced MYC expression in the siMYC treated HUVECs by TaqMan RT-PCR analysis. Data are expressed as Mean ± S.D.

The information on which the network was based comes from many cell types. Although we filtered and refined the network by only including genes that are present in the model, validating 17226 individual network edges is challenging. We explored the feasibility of using the present network model to address known biological phenomena as a pathway toward functional validation of the network. Examining the connectivity hierarchy, we found that oncogenes and tumor suppressor genes are among the highest ranked molecules (hub nodes) based on the number of connections in which they are directly engaged. For example, the number of connections that P53, SRC, and MYC directly engaged is 301, 243, and 173 respectively, ranking at the 2^nd^, 8^th ^and 16^th ^places in terms of connectivity in the interactome. This observation recapitulates the emerging role of oncogenes and tumor suppressor genes in angiogenesis regulation [1,5,23,24]. We focused our analysis on MYC by first dissecting its interactions with other genes in the network. A subnetwork centered on MYC was constructed from the initial interactome (Fig. 5A). It was found that MYC is linked to a variety of angiogenesis regulators with high connectivity within the network, suggesting a central role of MYC in angiogenesis regulation. We hypothesized that inactivation of MYC function would have profound angiogenic defects in this co-culture assay. We tested this hypothesis by attenuating MYC expression in HUVECs using c-MYC siRNA. Cord formation was significantly suppressed in siMYC treated cultures (Fig. 5B &5C). Cord mass, branching pattern, and responsiveness to exogenous VEGF were all inhibited, which is consistent with the network prediction. Knockdown of MYC expression by the siRNA was confirmed in parallel by TaqMan RT-PCR analysis (Fig. 5D). The involvement of TGF-β, the top node in the present angiogenesis network was also validated (data not shown).

### Assessment of compound antiangiogenic activity by network perturbation analysis

**Figure 6:**
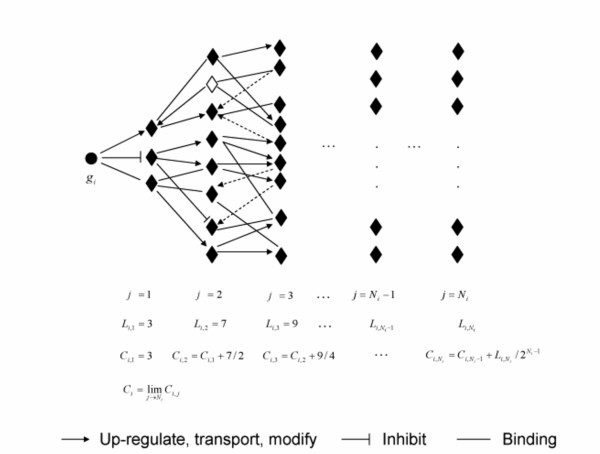
**Schematic diagram of the network algorithm to estimate connectivity index (*C*_*i*_) from the interactome**. Filled circles represent the i^th ^gene (*i *= 1,2, ..., *K*), and filled diamonds the other genes reported in the literature to have one or more functional associations with the i^th ^gene. Broken arrows are associations that were not taken into the calculation due to the reverse direction along the connection path. *L*_*i*,*j *_is the number of unique genes connecting to the i^th ^gene at the j^th ^step, *C*_*i*,*j *_is the connectivity of the i^th^gene at the j^th ^step, and *C*_*i *_is the connectivity of the i^th ^gene, which is the limit of *C*_*i*,*j*_.

**Figure 7:**
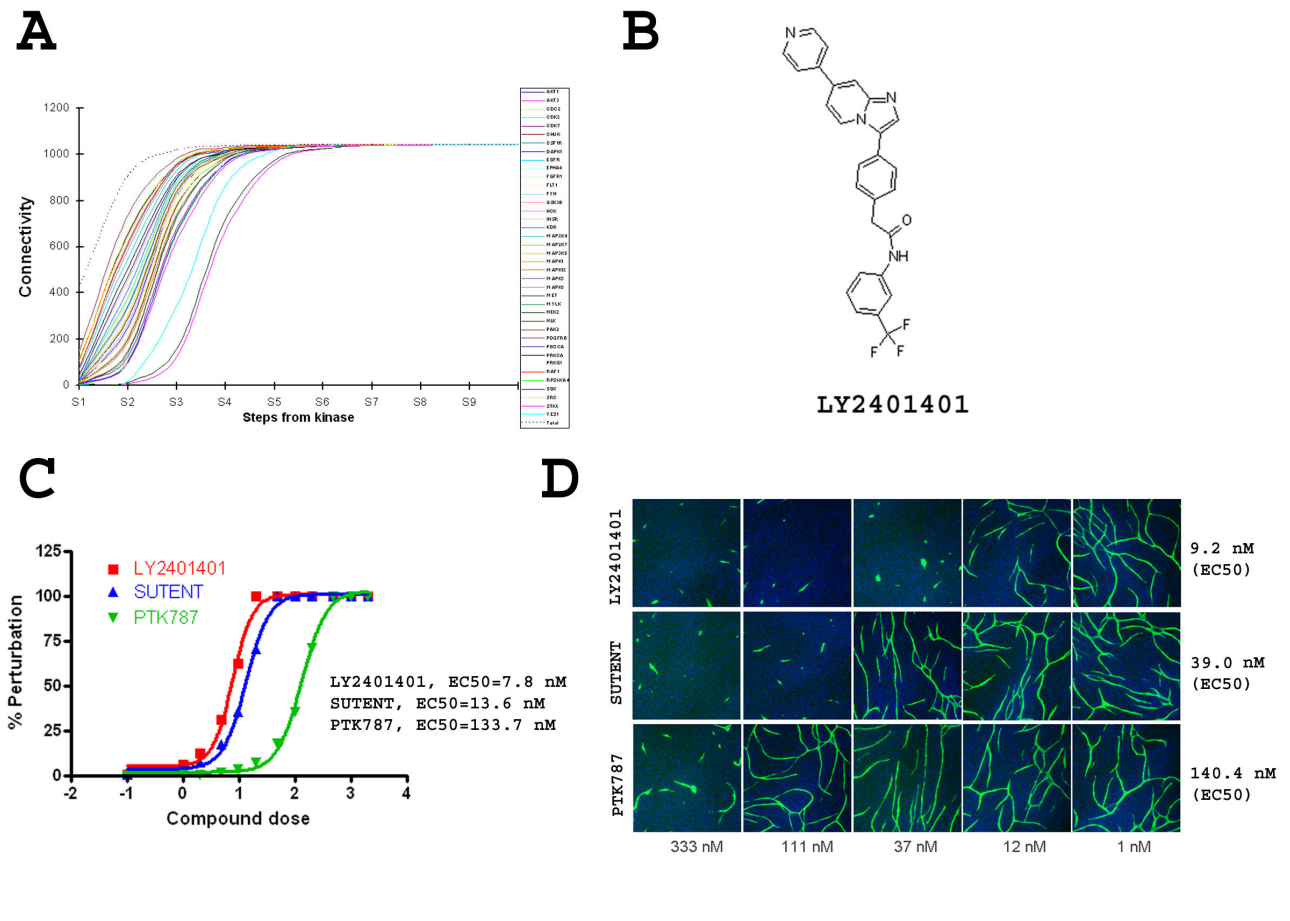
**Assessing compound phenotypic activity through network perturbation analysis**. **(A)**. Characteristic connection curves of 39 kinases mapped to the interactome. 39 kinases were mapped to the interactome; collectively, they directly connect to 525 genes in the network. A network walking algorithm was developed to estimate the spread of the connectivity of each kinase by calculating the total number of nonredundant genes it connected at each step along the connection pathway. The smoothed connectivity curve of each of the 39 kinases was shown, with the maximum reaching 1040 for each kinase. Summation of connectivity for 39 kinases is represented by the broken line. **(B)**. The chemical structure of LY2401401, a novel small molecule and a multi angiogenesis kinase inhibitor tested in the network perturbation experiment. **(C)**. The connectivity index was calculated by an exponentially weighted sum of the connectivity at each stage of each kinase. The dose dependent network perturbation of each small molecule inhibitor was simulated as described in the methods section. The data were imported into Graphpad Prism software to estimate the EC50 values of network perturbation using Sigmoidal dose-response curve fitting. **(D)**. Three small molecule kinase inhibitors were tested for cord formation. They all showed dose dependent inhibition of angiogenesis. Representative image summaries of the dose dependent responses are shown. The cord area data were imported to Graphpad Prism software, and normalized to the control group for EC50 calculation, using the same method as the one that was used to calculate the simulated network perturbation values. Shown in the figure are mean EC50s for each compound, averaged from two to four independent experiments.

We further explored the application of network analysis to understand compound polypharmacology. Since kinases have increasingly become effective targets for anti-angiogenic drug discovery [25-27], we assessed kinase contributions to angiogenesis regulation in the co-culture model by network analysis. First, 39 kinases were mapped to the interactome with a total of 525 directly-connected genes, accounting for 44% of the total number of genes in the refined interactome. Any mapped kinase is engaged in the rest of the network through multi-step connections. Accordingly, we developed a "network walking" algorithm to model the connection cascade of kinases within the network. The methods are described in the method section and shown in a schematic diagram in Fig. 6. Using this algorithm, the characteristic connection curves of the 39 mapped kinases were determined and are shown in Fig. 7A. All kinases were able to connect to the rest of the interactome (except for singletons), and the connectivity plateaus at the connection of 1040 within the interactome. This property was used to assess the potential maximal perturbation of the interactome if a given kinase is inhibited. While every connected molecule plateaus at the same number of connections, each arrives at a different rate, which can be assessed by the slope of the connectivity curve. Since the spread of activity along the network can not reach 100 percent penetration at each step, and the penetration is expected to decay dramatically at later steps due to multiple inputs and outputs at each node, we thus defined a perturbation index calculated from the data collected by the network walking algorithm using a weighted sum method as described in the method section.

Using network perturbation analysis to assess drug phenotypic activity, we tested three multi-targeted anti-angiogenic kinase (MAK) inhibitors: Sunitinib, PTK787, and LY2401401. The analysis was performed based on the number and the identities of the kinases that are inhibited by a given compound at its respective potency as described in the method section. Sunitinib and PTK787 have been previously tested in clinical trials [25], while LY2401401, is a novel Lilly MAK inhibitor with a distinct kinase activity profile. LY2401401 (Fig. 7B), an imidazopyridine, is an orally bioavailable, broad-spectrum inhibitor of the VEGF and PDGF receptors. Additionally, Flt3, Kit, Tie-2 and the Eph family of receptors are also sensitive to this small molecule inhibitor (SMI). To differentiate these MAK compounds, it is important to determine if the unique kinase profile of LY2401401 is advantageous to achieve anti-angiogenic efficacy. We simulated the potential network perturbation by the three molecules based on the integrated perturbation index of every kinase that a given SMI is active against. The resulting theoretical dose response curves were used to estimate the simulated EC50 of network perturbation (Fig. 7C). The compounds were ranked by their simulated activities as LY2401401>Sunitinib>PTK787, with the EC50 values estimated to be 7.8 nM, 13.6 nM, and 133.7 nM, respectively. The network analysis clearly differentiates these three MAK molecules. Interestingly, the network analysis predicted that Sunitinib would be far more potent than PTK-787, although their activities against KDR are rather close (49 nM, and 58 nM, respectively for PTK-787 and Sunitinib).

We then determined the actual angiogenesis activity of each compound by testing its dose dependent response directly in the cord formation assay. They were ranked in the same order as inferred by the network simulation (Fig. 7D). The observed EC50 values from multiple independent experiments were determined to be 9.2 nM (± 9.1 nM s.d.; n = 3), 39.0 nM (± 9.2 nM s.d.; n = 4), and 140.4 nM (± 53.7 nM s.d.; n = 2) for LY2401401, Sunitinib, and PTK787, respectively (Fig. 7D). The actual compound activity in the cord formation bioassay was consistent with the predicted values based on the network perturbation analyses.

## Discussion

Angiogenesis involves various cellular and multi-cellular events such as cell proliferation, survival, differentiation, migration, branching and sprouting. Such complicated processes are integrated via intrinsic and extrinsic regulatory mechanisms. Studying angiogenesis in the context of a global gene association network is critical to better understand angiogenesis and to improve cancer drug targeting strategies. For instance, through network analysis, peroxisome proliferative-activated receptor δ was recently identified as a "hub" node in a network that plays critical roles in mediating a tumor angiogenesis switch in human pancreatic cancers [28]. Over several decades, many proangiogenic factors have been uncovered, subsets of which are used by various malignant tumors preferentially [1,8]. However, the proangiogenic signals used by tumors are not static. It is thus unlikely to find a pleiotropic therapy by targeting a single proangiogenic factor. Moreover, tumors develop escape strategies by expressing alternative proangiogenic factors [13]. Unsurprisingly, mixed results have been observed in clinical trials with anti angiogenesis agents being used as a mono therapy or in combination therapies [27,29,30]. It has been well accepted in the field that targeting a single gene is not sufficient [31]. However, how to target multiple genes? What are the desirable combinations of targets? And how to achieve efficacy with less rebound possibility? These are some key issues to be addressed. In this light, a comprehensive gene association network governing angiogenesis is needed to aid rational anti-angiogenesis drug design.

In the present study, we first characterized an *in vitro *angiogenesis co-culture model using high content imaging analysis and found that cord formation is highly cell-passage dependent. We then profiled HUVEC and fibroblast cells at different passages, from which we identified more than two thousand genes whose expression was passage-dependent. Using a natural language processing algorithm, we retrieved a large collection of reported functional associations (binding, regulation, transport and modification) among identified genes. Analysis of the interactome indicated that it is functionally related to angiogenesis. Topologically, it displayed a typical characteristic of a scale-free network system, that is, 20% of genes account for 80% of functional connections. The interactome recapitulated the involvement of oncogenes in angiogenesis regulation, which was subsequently validated by siRNA mediated gene silencing. We further hypothesized that the interactome we constructed is essential and sufficient to support angiogenesis in the co-culture model system.

Anti-angiogenic efficacy from a given drug can be thought of as its ability to disrupt essential functional associations in the interactome. Conceivably, inhibitors capable of affecting more hub nodes should deliver faster and stronger network perturbation, eliciting enhanced anti-angiogenic efficacy. Therefore, we implemented a network walking algorithm to calculate connectivity indices in the interactome. The connectivity index for the *i*th gene is a quantity extracted from the interactome that measures its effectiveness in perturbing the entire angiogenesis interactome on inhibition. This is important as a way to address the important polypharmacology of anti-angiogenic compounds.

The present study takes kinase inhibitors as an example. Targeting multiple kinases is generally believed to be a viable antiangiogenic strategy and has been proven clinically [25-27,29]. Sunitinib and Nexavar (sorafenib), two small molecule kinase inhibitors, are currently registered therapies. However, PTK-787 failed to achieve statistical significance in clinical trials [32], although the exact mechanism accounting for the failure is not clear. Targeting the right spectrum of kinases by a single SMI is critical for the development of successful anti-angiogenic drugs [31]. Although many kinases are reportedly angiogenesis related, they may differ in downstream signalling events, crossreactivity, kinetics, etc. For a given small molecule kinase inhibitor, translation of its kinase profile to phenotypic activity is challenging. In the present study, we explored the feasibility of estimating the anti-angiogenic activity of a kinase inhibitor via network perturbation analysis. The current heuristic approach addressed the number and identity of the kinases that a given compound is active against as well as the respective potency. The simulation was conducted to estimate network perturbation by a novel MAK compound LY2401401 along with two clinically tested MAK compounds. The estimated anti-angiogenic activities for the three kinase inhibitors from the simulation were found to match well with their actual activities on separate bioassays (Fig. 7). Because the current network contains more than 17,000 edges, it would be desirable if the network could be further refined by more stringent filtering to potentially increase its predictive power. This might be done by refining the network using network connections inferred directly through comprehensive microarray studies, or directly generated protein-protein interaction data. In addition, the complexity of the current interactome, which contains data of different types, makes systematic filtering in an unbiased way difficult and should be considered for future studies.

Inferring a regulatory network from DNA microarray data remains a great challenge despite the fact that numerous computational methods have been published [33-39]. Biological processes are complex and involve hundreds of genes, thus requiring a large amount of diverse data. In addition, DNA microarray experiments only interrogate transcriptionally-regulated genes, so it is inevitable that a significant portion of false positive and false negative interactions in an inferred network will be generated. In the present study, instead of directly inferring a regulatory network, we constructed an interactome from differentially expressed genes by applying a literature text mining technique to extract functional associations that had been observed from experiments; thus we anticipate fewer false positives. With the shortest path approach, we could recover functional associations among genes where the expression is not transcriptionally regulated, thus we anticipate fewer false negatives. For example, 20% of the genes constituting our interactome were not differentially expressed either in the HUVEC or NHDF cells, yet they ranked rather highly in the connectivity hierarchy. Since the natural language processing of literature text can be error-prone, the interactome we constructed has its own limits. The current approach is limited to address the complexity of *in vivo *cancer angiogenesis which not only involves multiple cell type and signalling pathway interactions, but the interactions are also geometrically and temporally restricted. For example, anti-angiogenic drug-induced inhibition of vessel formation subsequently causes changes in blood flow and creates hypoxic regions in the tumor. Such changes are highly heterogeneous and can be further compounded by many other human factors. Although HIF is one of the key nodes identified in our interactome, applying network analysis to address *in vivo *angiogenesis is still challenging. Moreover, the current interactome is a collection of functional associations among participating genes without the inclusion of feedback regulatory mechanisms; it is not suitable for an advanced dynamic or kinetic analysis. For these reasons, we took advantage of a general statistical property of a biological network, namely, the characteristics of a scale-free network system [22]. The network walking algorithm captures unevenly distributed connectivity among functionally associated genes in the constructed angiogenesis interactome and uses that information to calculate how much and how fast an external perturbation of the network can be induced by a kinase inhibitor. This was then used to estimate potential potency of the inhibitor for its anti-angiogenesis effect. A near perfect match between estimated and measured potencies proves the statistical properties of the interactome are informative for network perturbation analysis. It would be also very interesting to extend our current network approach to other angiogenesis related data sets, such as the data generated from a time course study, as well as applications beyond angiogenesis in the future. In summary, our current effort defined a comprehensive gene functional association network and demonstrated the feasibility of assessing drug phenotypic activity through network perturbation analysis. Corroborative studies with a larger pool of compounds, refined network parameters and an improved modelling approach would be warranted in further investigations.

## Conclusion

We characterized an *in vitro *angiogenesis co-culture model via high content imaging analysis. We determined that HUVEC competence and NHDF supportiveness of cord formation are highly cell-passage dependent. We built a comprehensive gene association network to model angiogenesis regulation via complementary profiling of passage dependent gene expression in two cell types that constituted the co-culture model. We developed a novel algorithm to extract connectivity information from the gene functional association network. Using multi-targeted anti-angiogenic kinase inhibitors as an example, our data indicate the feasibility of applying network perturbation analysis to assess drug polypharmacology and phenotypic activities.

## Methods

### Reagents and Cells

Recombinant VEGF was purchased from R&D Systems (Minneapolis, Minnesota). Human umbilical vein endothelial cells (HUVECs) and Normal Human Dermal Fibroblast (NHDF) cells and culture medium were purchased from Cambrex (Walkersville, Maryland). Cells were maintained in EGM with 10% FBS and FGM-2, respectively for HUVECs and NHDFs, according to manufacture's instructions. *In vitro *angiogenesis co-culture AngioKit and Optimized medium were purchased from TCScellworks (Buckingham, UK).

### *In vitro *angiogenesis assay

*In vitro *HUVEC cord formation co-culture was performed using the AngioKit commercially available from TCScellworks (Buckingham, UK) adapted to a 24-well format. To test HUVEC and NHDF passage dependence effects, the co-culture was done in house adapted to a 96-well format. Briefly, HUVEC cells were co-cultured onto human dermal fibroblast feeder layers in Optimised medium (TCScellworks, Buckingham, UK) for a total of about 12 days. Treatment started the day when the plates were received; usually three to four days after initial plating. Medium with or without growth factors or compounds was replenished every 2 to 3 days. Cells were fixed with 70% cold ethanol at the end of the culture and processed for anti-CD31/Alexa 488 immunofluorescence. To visualize feeder layer fibroblasts, some cultures were stained using a polyclonal antibody against Vimentin (Abcam, Cambridge, Massachusetts) along with an Alexa 568 conjugated secondary antibody to examine the feeder cell integrity. Nuclear staining was performed using Hoechst staining (Invitrogen Molecular Probe, Carlsbad, California).

Immunofluorescence stained co-culture plates were scanned using ArrayScan VTI (Cellomics, Pittsburgh, Pennsylvania) and the multi-parameter tube formation Bioapplication was used for quantification. Morphological measurements included cord length, width, area, percent connected cord, branching node and segment count, nuclei per cord, angiogenic index (expressed as the percentage of area covered by connected tube), etc. General cytotoxicity was monitored by examination of the nuclear staining of the feed layers.

### Attenuation of gene expression by siRNA

HUVECs were trypsinized by 0.05% Trypsin/EDTA (Invitrogen, Carlsbad, CA), and 1 × 10^6 ^cells were nucleofected with siMYC (200 pmol each, Ambion, Austin, TX) using a nucleofector device (Amaxa, Gaithersburg, MD) adopting optimized A-034 protocols for HUVECs according to the instructions from the manufacturer. Transfected cells were appropriately diluted and plated in 96-well plates onto the NHDF feeder cells for the initiation of the cord formation assay. Cells were also plated in parallel into 6-well plates and collected in Trizol (Invitrogen, Carlsbad, CA) 48 h or 72 h later for total RNA extraction.

### Real Time RT-PCR

Total RNAs were extracted from Trizol and cleaned by RNeasy mini kit (Qiagen, Chatsworth, CA). Genomic DNA contamination was removed by DNA-free kit (Ambion, Austin, TX), and cDNA synthesis was done by using a high capacity cDNA archive kit (Applied Biosystems, Forster City, CA). The cDNAs were used as templates for real-time PCR with a universal PCR master mix (Applied Biosystems, Foster City, CA). Real time PCR was performed on a ABI 7900 HT. The average values were normalized to 18s rRNA. Delta CT method was applied to calculate the relative gene expression level [40].

### RNA isolation and microarray experiments

RNA isolation and microarray studies were performed as described previously [41]. Briefly, HUVECs and NHDFs with different passages were cultured to about 80% confluency. RNA was isolated using Trizol™ (Life Technologies, Inc. Invitrogen, Carlsbad, California) according to manufacture's instructions, and followed by cleanup using RNeasy spin columns (Qiagen, Valencia, California). RNA labelling was performed on the Onyx robotic system from MWG-Biotech and Aviso using the standard labelling protocol. Biotin labelled cRNA was fragmented and hybridized to human whole genome U133 plus 2 GeneChip (Affymetrix, Santa Clara, California). Chip processing, imagine capturing, and raw data analysis were performed using the Affymetrix Microarray Suite MAS with default parameters.

### Statistical analysis to identify differentially expressed genes

Hybridized microarrays were analyzed by the Affymetrix microarray analysis software microarray suite 5 (MAS 5). The chip signals were normalized using the default method in Affymetrix MAS5, but setting the target at 2%-trimmed mean to 1500 instead of 500. HUVEC were analyzed with four passages. We designated the cell passages 5, 8, 12 and 22 as 1, 2, 3 and 4 respectively. For each probeset, the signal data were fitted to a linear regression model of time to identify consistent gene expression changes over cell passage. Three batches of the NHDF cells were analyzed, including early passage neonatal NHDF, early passage adult NHDF, and late passage adult NHDF cells. To reduce a possible batch to batch effect, the MAS 5 signals for each chip were re-normalized by Local Polynomial Regression Fitting (loess) approach using one neonatal sample as the baseline on a log scale. The re-normalized signal data were fitted to an ANOVA model to identify differential gene expression changes between the different fibroblast cells.

To control for a false positive rate of testing the expression change of thousands of genes simultaneously, the false discovery rate (fdrate, or FDR) was estimated using an algorithm derived from Benjamini and Hochberg [42]. Probesets with a false discovery rate of 0.2 or smaller were considered as significant and followed up for further analysis. We excluded probesets called "absent" in all chips in a data set. These probesets usually have very low expression signals. In addition, we used expression fold change between different treatment conditions to filter out genes to make sure the gene expression changes are biologically meaningful.

PCA analysis was performed in R using the principal component analysis function with standardized data [43]. The first three principal components with > 80% variations were exported into Spotfire (Sommerville, MA) to aid in creating sample scatter plots. Hierarchical clustering analysis of DE genes was done in Spotfire with Euclidean distance by the complete linkage method.

To identify biological processes that were significantly changed with passages, the identified DE genes were analyzed using the DAVID functional annotation system (National Institute of Allergy and Infectious Diseases, NIH) [21]. Significantly changed biological processes were determined by the enrichment test provided by the DAVID system. To control for a false positive rate of testing the expression change of thousands of genes simultaneously, the false discovery rate (fdrate, or FDR) was estimated using an algorithm derived from Benjamini and Hochberg [42].

### Construct angiogenesis related interactome and calculate connectivity index

A tentative interactome was constructed using PathwayAssist from the union of the differentially expressed genes identified either from the HUVEC or the NHDF array experiment. PathwayAssist was configured to retrieve five different types of relationships defined in the software, namely, binding, expression, molecular transport, protein modification and regulation. The software introduced non-differentially expressed genes into the interactome in order to establish the shortest connection pathway between any two differentially expressed genes in the initial input gene list. This is an informative function since it could recover potentially non-transcriptional regulatory relationships between two or more genes. We cleaned up this initial interactome by removing genes that were not detected by either array study. In addition, genes that could not connect to any other genes were called orphans and removed from the interactome. Thus, the final version of the interactome we constructed can be described as follows:

Let *G *be the set of *K *genes in the interactome *I*, and *R *be one of the five relationships: binding, molecular transport, protein modification, inhibition or up-regulation. Thus, the interactome *I *we built can be formally described as

(1) *I *= {(*g*_*i*_, *g*_*i*_) ∈ *R *| *g *∈ *G; i*, *j *= 1,2, ... *K*}

We assumed *R *is transitive, that is, if gene A regulates gene B, and gene B regulates gene C, it is assumed that gene A regulates gene C. Except for binding among the five types of relationships, molecular transport, protein modification, up-regulation and inhibition are unidirectional.

We wrote a "network walking" algorithm (Figure 6) in a recursive function with which we calculated the number of unique genes, *L*_*ij*_, connecting to the i^th ^gene at the j^th ^step along the connection path in the interactome. We define connectivity of the i^th ^gene at the j^th ^step along the connection pathway as

(2) *C*_*i*,*j *_= *C*_*i*,*j*-1 _+ *L*_*ij*_/2^*j*-1^

Thus, for the i^th ^gene in the interactome its connectivity at the j^th ^step in its connection pathway is the connectivity at the (j-1)^th ^step plus weighted number of unique genes at the j^th ^step. We assigned 1/2^*j*-1 ^as an arbitrary weight to the number of unique genes connecting to the i^th ^gene at the j^th ^step, that is, the weight exponentially decreases with the number of steps away from the i^th ^gene. Obviously *C*_*i*,*j *_is a non-decreasing function. We further define connectivity of the i^th ^gene *C*_*i *_as

(3) $$C_{i}=\underset{j\to N_{i}}{\lim}C_{i,j}$$

where *C*_*i *_is the plateau of connectivity of the i^th ^gene and *N*_*i *_the number of steps *C*_*i*,*j *_takes to arrive at its limit or the plateau. Since every gene in the interactome is connected, the total number of genes a gene connects to is the same, that is, after *N*_*i *_steps the connectivity of the i^th ^gene will arrive at its plateau. However, they differ in the number of steps *N*_*i *_or the rate of the arrival at the plateau. Some genes may take more steps than the others to arrive at the platueas, depending upon what and how many genes it connects, especially at early steps. Schematic elucidation of the algorithm is shown in Fig. 6.

### Simulation of network perturbation

Let *S *be a small molecule kinase inhibitor, and *P*_*s*, *D *_be the level of network perturbation by the small molecule at a hypothetical dose *D*. *P*_*s*, *D *_is estimated as

(4) $$P_{s,D}=\sum_{i=1}^{K}D\times C_{i}/2\times IC50_{i,s}$$

where *K *is the number of kinases in the interactome *I *that can be inhibited by the small molecule *S*, *IC*50_*i*,*s *_is the IC50 value of the small molecule *S *against the i^th ^kinase. Their relative IC50 values against various kinases were measured at Upsate (UBI) or in house. To simulate the dose response curve, we set the theoretical dose D from 0.1 nM to 2 μM. If a term in (4) is more than the maximum number of the connected genes, the term is then set to the plateau. Summed network connectivity was used to calculate the percent perturbation with the plateau to be set at 100% network perturbation. The resulted simulated dose dependent network perturbation was then imported into the program GraphPad Prism to estimate EC50 of network perturbation by the small molecule *S *by performing Sigmoidal dose-response curve fitting.

## Abbreviations

DE: differential expression; GO: Gene Ontology; HUVEC: human umbilical vein endothelial cell; MAK: multi-targeted anti-angiogenic kinases; NHDF: normal human dermal fibroblast; PCA: principle component analysis.

## Authors' contributions

JS conceived the project. YC, TW, JMY, and JS designed the study. YC, FL, and LY performed the experiments. TW contributed to bioinformatics analyses and algorithm development. H–RQ performed the statistical analysis. TW, TPB, JJS, JMY, and JS interpreted the data. TW, JMY, YC, and JS wrote the manuscript. All authors read and approved the final manuscript.

## Supplementary Material

Additional file 1 This table lists the 1103 differentially expressed genes identified in HUVECs. Click here for file

Additional file 2 This table lists 787 non-redundant common differentially expressed genes identified in NHDF cells. Click here for file

Additional file 3 This file lists the detailed information about the interactome. Click here for file
